# Antiurolithiatic Potential of Three Sri Lankan Medicinal Plants by the Inhibition of Nucleation, Growth, and Aggregation of Calcium Oxalate Crystals *In Vitro*

**DOI:** 10.1155/2022/8657249

**Published:** 2022-04-12

**Authors:** Sasindu Punyamali Hewagama, Ruwani Punyakanthi Hewawasam

**Affiliations:** ^1^Department of Medical Laboratory Science, Faculty of Allied Health Sciences, University of Ruhuna, Galle 80000, Sri Lanka; ^2^Department of Biochemistry, Faculty of Medicine, University of Ruhuna, Galle 80000, Sri Lanka

## Abstract

**Background:**

Deposition and formation of stones in any part of the urinary system is called urolithiasis. CaOx is the predominant component of most stones, and the formation of these stones is a multistep process that includes supersaturation, nucleation, aggregation, growth, and retention. In ayurvedic medicine, medicinal plants are used for the management of kidney stones. The objective of this study was to determine the effect of aqueous, ethanol, and hexane extracts of *Drymoglossum piloselloides* leaves, *Kalanchoe laciniata* leaves, and *Aegle marmelos* flowers against CaOx urolithiasis *in vitro*.

**Methods:**

The crystallization of CaOx monohydrate (COM) and dihydrate (COD) was induced in a synthetic urine system. The nucleation, growth, and aggregation of crystals were measured using spectrophotometric methods. The results were compared against the polyherbal drug, Cystone, under identical concentrations. Crystals generated in the urine were also observed under light microscopy. Statistical differences and percentage inhibitions were calculated using standard formulae and compared. A preliminary phytochemical screening was also performed to detect active phytoconstituents present in the three plants used in the study.

**Results:**

The results obtained clearly demonstrated that *Kalanchoe laciniata*, *Aegle marmelos*, and *Drymoglossum piloselloides* have the capacity to inhibit the nucleation, growth, and aggregation of CaOx crystals. Microscopic examination of crystals revealed the presence of more COM than COD crystals but a dose-dependent reduction in crystals was observed in the presence of plant extracts. Hexane, ethanol, and aqueous extracts of all three plants had different capabilities to inhibit nucleation, growth, and aggregation of CaOx crystals but their activities were different at different concentrations. Preliminary phytochemical screening revealed the presence of reducing sugars, proteins, flavonoids, tannins, and polyphenol compound in *Kalanchoe laciniata* and *Drymoglossum piloselloides* and reducing sugars, proteins, anthracene glycosides, and saponins in *Aegle marmelos*.

**Conclusions:**

This study provided evidence that *Kalanchoe laciniata*, *Aegle marmelos*, and *Drymoglossum piloselloides* have the potential to be developed as inhibitors of nucleation, growth, and aggregation of CaOx crystals in the treatment of urolithiasis.

## 1. Introduction

Urolithiasis has become the third most common affliction of the urinary tract affecting approximately 12% of world's population with an increasingly high recurrence rate in males than in females [1]. Aetiology is multifactorial and is strongly influenced by biochemical, epidemiological, and genetic risk factors [2]. Calcium oxalate (CaOx) is the predominant component of most stones accounting for more than 80% of stones. Remaining 20% are mainly composed of struvite, cysteine, and uric acid [3]. The pathogenesis of urolithiasis is a multistep process that results from a number of physicochemical events. The crystallization of CaOx begins with an increased urinary supersaturation, with the subsequent formation of solid crystalline particles within the urinary tract. It is followed by nucleation, by which stone-forming salts in the supersaturated urinary solution coalesce into clusters that then increase in size by the addition of new constituents [4]. These crystals then grow and aggregate with other crystals in solution and are ultimately retained and accumulated in the urinary tract. Renal injury promotes crystal retention and the development of a stone nidus on the renal papillary surfaces and further supports crystal nucleation at lower supersaturation level [2]. Supersaturation of urine with mineral salts is the major cause of urolithiasis. The level of urinary supersaturation correlate with the type of stone formed. Therefore, the reduction of supersaturation is effective in the prevention of stone recurrence [3].

Management of urolithiasis depends on the size and location of the stones. Thiazide diuretics and alkali citrate are used commonly in the prevention of recurrence of urolithiasis. Stones larger than 5 mm or stones that fail to pass through should be treated using interventional procedures, such as endoscopic stone removal, extracorporeal shock wave lithotripsy (ESWL), ureteroscopy (URS), or percutaneous nephrolithotomy (PNL). These procedures are very expensive for many patients and are associated with recurrence of kidney stones, which is often up to 60%. They also require careful follow-up for many years for possible complications such as acute renal injury [2]. Therefore, in many countries, including Sri Lanka, phytotherapeutic agents are widely used as complementary and alternative therapies for the management of urolithiasis.

Medicinal plants have played a significant role in various ancient traditional systems for the management of kidney stones. These plants provide a cheap source of drugs and are regarded as comparatively safe with minimal or no side effects, are readily available, and are affordable [3].

*In vitro* systems for experimental nephrolithiasis are widely used to differentiate the systems that investigate the physical chemistry of stone formation or systems that explore the pathophysiology of renal stone disease. To serve the first purpose, *in vitro* crystallization systems that study processes of crystal nucleation, growth, and aggregation are widely used [5].

Various medicinal plants have been reported to inhibit CaOx crystallization. Atmani and Khan [6] have reported that an extract from the herb *Herniaria hirsuta* L., a plant that is traditionally used in Morocco for the treatment of lithiasis, promoted the nucleation of calcium oxalate crystals by increasing their number but decreasing their size. Later they demonstrated that *H. hirsuta* blocked crystal binding to cultured renal cells [7]. Similar effects were observed on calcium oxalate crystallization *in vitro* for the aqueous extract from *Phyllanthus niruri* L., a plant that is used in traditional Brazilian medicine for the treatment of urolithiasis [8]. The authors reported that the extract interfered with the CaOx crystallization process by reducing CaOx crystal growth and aggregation [8]. Garimella et al. [9] demonstrated that an extract prepared from the seeds of *Vigna unguiculata* (L.) Walp. that is used in traditional Indian ayurvedic medicine inhibited the precipitation of calcium and phosphate *in vitro*. Several traditional Chinese medicines (TCM) or plants that are used in Kampo medicine also have demonstrated their abilities to inhibit calcium oxalate crystallization [10, 11]. Overall, the *in vitro* crystallization experiments confirmed that prophylaxis of renal stones could be achieved by reducing supersaturation through promotion of small crystal nucleates.

**Figure 1:**
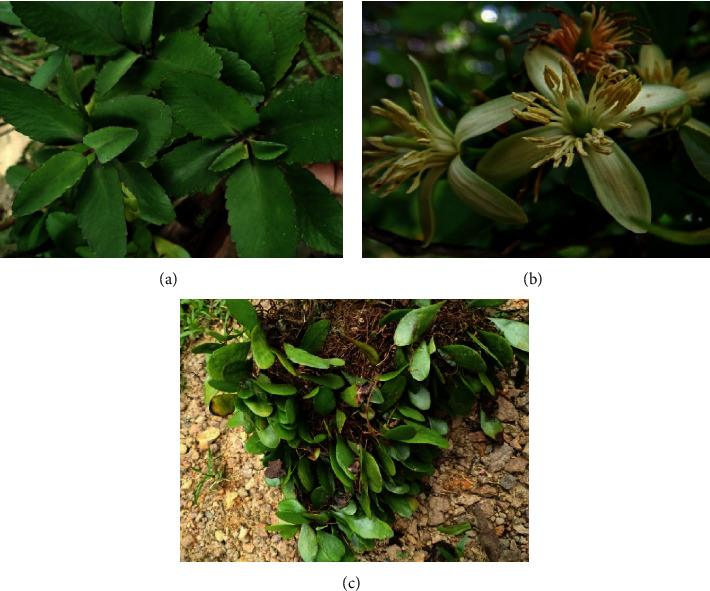
Three plants, *Kalanchoe laciniata* leaves (Crassulaceae, “Akkapana”) (a), *Aegle marmelos* flowers (Rutaceae, “Beli”) (b), and *Drymoglossum piloselloides* leaves (Polypodiaceae, “Kaasi pethi”) (c) are shown with their respective family name, local name, and the plant part used in the study.

Out of the three plants used in this study, *Kalanchoe laciniata*, commonly known as “Akkapana” (Figure 1(a)), is a succulent, glabrous herb with stems stout, slightly branched, leaves bi pinnatifid, petiole-like base, inflorescence 12–25 cm long, cymose, paniculate cymes with many flowers, flowers bright chrome yellow, pedicel 6–9 mm long, fruit of 4 follicles, seeds numerous [12]. *Aegle marmelos*, which is commonly known as “Beli” (Figure 1(b)) in Sri Lanka, is a widely consumed fruit grown in home gardens. It is a tree of about 10–13 m in height with short, strong, sharp, and spiny branches. It has alternate leaves, trifoliate or 1-2 leaflets, inflorescences axillary, 4-5 cm long, clustered, flowers in racemes, petals white, glandular, fruits globose, with a hard woody shell, numerous seeds in a clear, sticky, and edible pulp [12]. *Drymoglossum piloselloides,* which is commonly known as “Kasi pethi” (Figure 1(c)) in Sri Lanka, is an epiphytic fern climbing on branches of trees and shrubs, rhizomes long, slender, covered with depressed peltate laciniated scales, sori arranged in a broad, continuous, and marginal lines often confluent and covering whole undersurface, capsules mixed with a few stellate paraphyses [12]. All three plants are used by the traditional ayurvedic medical practitioners in Sri Lanka for the treatment of urolithiasis. In addition to the antiurolithiatic activities, *Aegle marmelos* is used to treat dysentery, dyspepsia, malabsorption, neurological diseases, edema, vomiting, and rheumatism [13]. *Drymoglossum piloselloides* leaves are used in the treatment of bacterial infections, cough, gonorrhea, eczemas, and haemorrhage [14]. *Kalanchoe laciniata l*eaves are reported to have medicinal properties in the treatment of ulcers, gastritis, inflammation, fever, cough, and so on [15].

Cystone is a polyherbal formulation developed specifically for the management of urolithiasis. This formulation has been widely used in the clinical practice for many years and was approved by the regulatory authorities in India as an ayurvedic formulation for the treatment of urinary calculi [16]. Therefore, it is widely used as a natural supplement in the prevention of urolithiasis and as a reference drug in many experimental studies for the screening of medicinal plants for antiurolithiatic potential. Since different phytochemicals are extracted to solvents with different polarities, the objective of the present study was to determine the *in vitro* antiurolithiatic potential of hexane, ethanol, and aqueous extracts of leaves of *Drymoglossum piloselloides*, leaves of *Kalanchoe laciniata*, and flowers of *Aegle marmelos* on nucleation, aggregation, and growth of CaOx crystals compared to the widely used polyherbal supplement, Cystone, and to justify the scientific basis for the use of these plants in traditional systems of medicine.

## 2. Materials and Methods

### 2.1. Plant Material

Three plants, *Drymoglossum piloselloides*, *Aegle marmelos*, and *Kalanchoe laciniata*, were collected from Galle in the Southern province, Sri Lanka, located within 6° 3′ 13″ of north latitude and 80°12′ 42″ of east longitude. Botanical identity was determined by the descriptions given by Jayaweera [12]. *Drymoglossum piloselloides* leaves, *Aegle marmelos* flowers, and *Kalanchoe laciniata* leaves were washed thoroughly with water and allowed to dry. They were cut into small pieces and then dried until a constant weight is reached at 40°C in an oven. The dried materials were coarsely ground and stored in an air-tight container at 4°C.

### 2.2. Drugs and Chemicals

Cystone, the polyherbal drug, was purchased from Himalaya Drug Company. All solvents and chemicals used in this experiment were commercially available and of reagent grade.

### 2.3. Preparation of Plant Extract

10 g of each plant material was soaked in 100 ml of absolute ethanol and hexane, respectively. Volumetric flask was properly sealed and was left for 4 days in a shaking water bath. The mixture was filtered using filter paper and the solvents were evaporated using a rotary evaporator. The weight of crude extracts was measured and dissolved in a minimum amount of 100% DMSO to prepare the stock solution. Extracts were stored in an air-tight bottle at 4°C for further use.

To prepare aqueous extract, 2.5 g of dried plant material was mixed with 60 ml of distilled water. Then, it was refluxed for 2 hrs. The extract was filtered and dried to get crude extract and it was dissolved in a minimum amount of 100% DMSO. Final extracts were stored at 4°C for further use. Concentration of each plant material was calculated and the respective concentration series was prepared in distilled water to carry out the nucleation, growth, and aggregation assays.

### 2.4. Phytochemical Screening

Preliminary phytochemical screening for plants under study was carried out. It was a qualitative analytical study that aimed to find phytoconstituents present in the three plants. Extracts were prepared according to the respective test methods. The extracts were subjected to a qualitative analysis to detect major classes of phytoconstituents, reducing sugars, proteins, flavonoids, tannins, saponins, anthracene glycosides, polyphenol compounds, cyanogenic glycosides, and alkaloids, as described by Evans and Sofowora [17, 18].

### 2.5. Determination of Antiurolithiatic Activity Using Nucleation, Growth, and Aggregation Assays

Different stages of the formation of CaOx crystals were studied using *in vitro* methods with or without the plant/drug extract, which was determined by nucleation assay, growth assay, and aggregation assay.

#### 2.5.1. Nucleation Assay

The stone formation initiates with the occurrence of nuclei. The inhibitory activity of the extracts (200, 400, 600, 800, and 1000 *μ*g/mL) on the nucleation of CaOx crystals was determined by a spectrophotometric assay as described by Bawari et al. with slight modifications [19]. Solution of calcium chloride (CaCl_2_) and sodium oxalate (Na_2_C_2_O_4_) were prepared at the final concentrations of 5 mmol/L and 7.5 mmol/L, respectively, in a buffer containing Tris (0.05 mol/L) and NaCl (0.15 mol/L) at pH 6.5. 1 ml of each concentration was mixed with 1 ml CaCl_2_ solution followed by the addition of 1 ml Na_2_C_2_O_4_ solution. Final mixtures were incubated for 30 min at 37°C. The optical density (OD) of the mixtures was measured at 620 nm with an UV-visible spectrophotometer (UV-1800 240 V, Japan). Percentage inhibition of nucleation was calculated using the following formula [19].

(1) $$\begin{array}{c} \%\text{ Inhibition}=\left[1-\frac{\text{OD Test}}{\text{OD Control}}\;\right]\times 100, \end{array}$$

where % Inhibition is percentage of inhibition, OD Test is optical density with plant extract/standard drug, and OD Control is optical density without plant extract/standard drug.

CaOx crystallization was observed under a light microscope in the presence and absence of extracts.

#### 2.5.2. Growth Assay

The growth of CaOx crystals was examined with or without the plant extract/standard drug following the method of Bawari et al. [19]. 4 mM CaCl_2_ solution and 4 mM Na_2_C_2_O_4_ solution (1 ml each) were added to 1.5 ml of solution containing NaCl (90 mM) buffered with Tris-HCl (10 mM) at pH 7.4. To this 30 *μ*l of CaOx crystal slurry (1.5 mg/ml CaOx slurry was prepared in a 50 mM sodium acetate buffer at pH 5.7) was added. The growth of CaOx crystals was then determined by measuring the rate of oxalate depletion from the solution at 214 nm wavelength for 600 s. The effect of each concentration of extracts on crystal growth was determined by the addition of 1 ml of extract (100 *μ*g/mL, 500 *μ*g/mL, and 1000 *μ*g/mL) to the reaction mixture and change in the optical density was recorded with an UV-visible spectrophotometer (G10S UV-Vis). Percentage inhibition of crystal growth was calculated.

(2) $$\begin{array}{c} \text{Relative inhibitory activity}\left(\%\right)=\left(\frac{C-S}{C}\right)\times 100, \end{array}$$

where *C* is the rate of reduction of free oxalate without any extract and *S* is the rate of reduction of free oxalate in the presence of extract.

#### 2.5.3. Aggregation Assay

When the crystals in solutions stick together, they form large particle aggregates. The inhibition of aggregation in the presence of the extract was evaluated using the method of Bawari et al. [19]. CaCl_2_ and Na_2_C_2_O_4_ solutions (50 mmol/l each) were mixed together, heated to 60°C in a water bath for 1 h and then incubated overnight at 37°C to prepare seed CaOx crystals. After drying, CaOx crystal solution (0.8 mg/ml) was prepared in a 0.05 mol/l Tris-HCl and 0.15 mol/l NaCl buffer (pH 6.5). 1 ml of extract (200, 400, 600, 800, and 1000 *μ*g/mL) was added to 3 ml CaOx solution, vortexed, and then incubated at 37°C for 30 min. Optical density of the final mixtures was read at 620 nm wavelength and percentage inhibition of aggregation was calculated as described in Section 2.5.1. CaOx crystal aggregation was also observed under the light microscope in the presence and absence of extracts.

### 2.6. Statistical Analysis

Each test was carried out in duplicate and data was expressed as mean ± SEM (standard error of the mean). For the statistical analysis of the data, group means were compared with one-way analysis of variance and post hoc analysis (SPSS 26.0 software). Dunnett's post hoc test was applied to identify significance among groups; *p* < 0.05 was considered to be statistically significant.

## 3. Results

### 3.1. Phytochemical Screening

The preliminary phytochemical screening revealed the presence of reducing sugars and proteins in all three plant extracts. Flavonoids, tannins, and polyphenol compounds were present in all plant extracts except *Aegle marmelos*. Anthracene glycoside and saponins were present only in *Aegle marmelos*. Alkaloids and cyanogenic glycosides were absent in all three extracts (Table 1).

### 3.2. Determination of Antiurolithiatic Activity Using Nucleation, Growth, and Aggregation Assays

#### 3.2.1. Nucleation Assay

**Figure 2:**
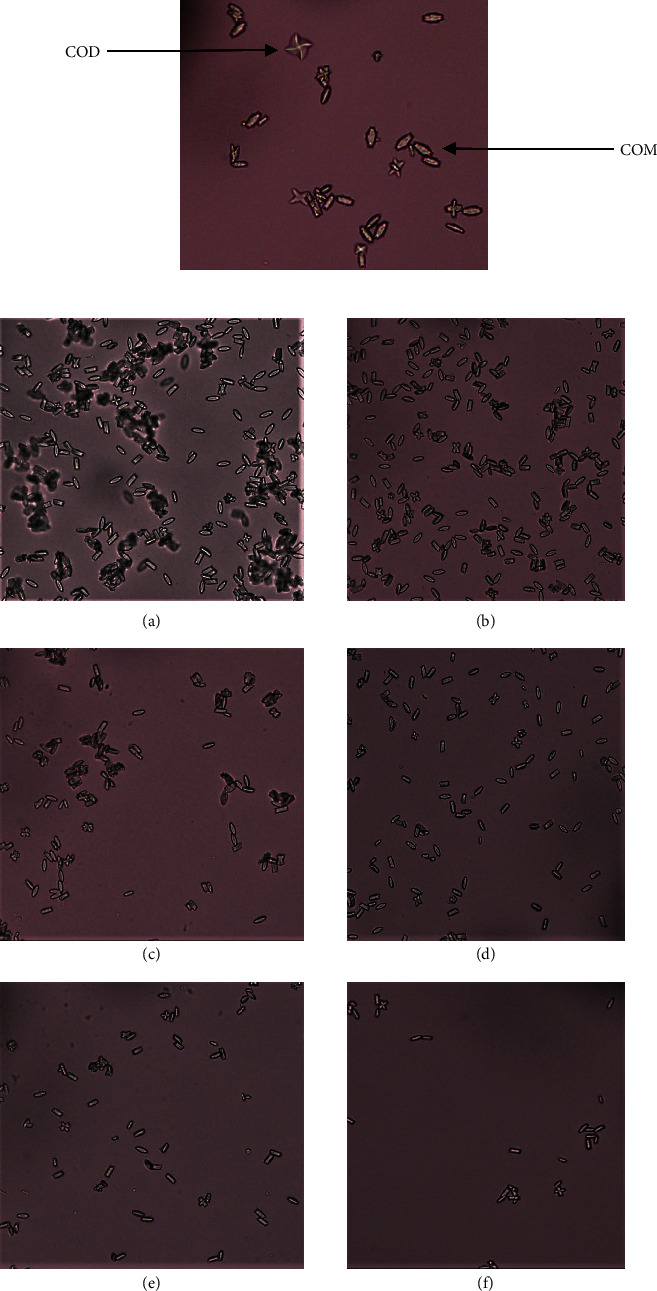
Representative micrographs of calcium oxalate crystals in the nucleation assay as observed under the light microscope (x40 objective) in the absence of the extract (a) and in the presence of ethanol extract of *Aegle marmelos* (EEAM) 200 *μ*g/mL (b), 400 *μ*g/mL (c), 600 *μ*g/mL (d), 800 *μ*g/mL (e), and 1000 *μ*g/mL (f).

The *in vitro* inhibitory effect of the three plant extracts on various phases of CaOx crystallization was determined by the time course of turbidity measured in synthetic urine at a series of concentrations and it resulted in the formation of numerous CaOx crystals. Microscopic photographs showed the formation of both types of CaOx crystals, biconcave, oval, and dumb bell-shaped COM and envelope-shaped or octahedral COD (Figure 2). Majority of crystals formed were of COM type.

**Figure 3:**
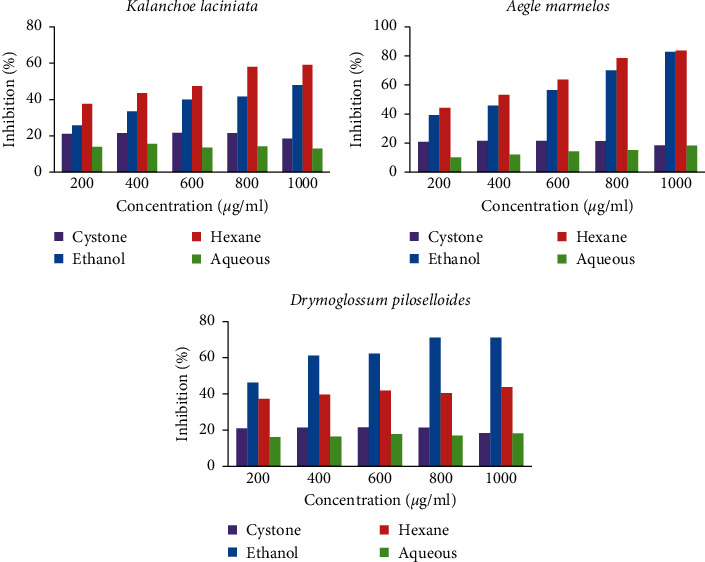
Percentage of inhibition of different extracts of plants on calcium oxalate nucleation. Each graph shows the dose response effect of each plant extracted in ethanol, hexane, and water against the standard drug, Cystone. Except for the aqueous extract of *Aegle marmelos* (AEAM) and aqueous extract of *Drymoglossum piloselloides* (AEDP) at the highest concentration of 1000 *μ*g/mL, all other extracts at all concentrations showed a significant difference in inhibition (*p* < 0.05) compared to the standard drug, Cystone.

The inhibition of CaOx crystal nucleation by Cystone and the inhibition of CaOx crystal nucleation by the extracts of *Kalanchoe laciniata* are presented in Figure 3 and Supplementary Table S1. Ethanol extract of *Kalanchoe laciniata* (EEKL) and hexane extract of *Kalanchoe laciniata* (HEKL) showed a dose-dependent inhibition on CaOx nucleation. The maximum inhibitory effect (59.01 ± 0.31%) was shown by HEKL at the concentration of 1000 *μ*g/mL, compared to the other extracts of the same plant which was higher than the standard drug. Interestingly, Cystone was less effective than these two extracts, but aqueous extract of *Kalanchoe laciniata* (AEKL) exhibited less inhibitory activity than the standard drug and the other two plant extracts. The values are depicted as mean ± SEM. Ethanol extract of *Aegle marmelos* (EEAM) and hexane extract of *Aegle marmelos* (HEAM) also showed a dose-dependent inhibitory effect. The highest percentage of inhibition was determined by the HEAM at the concentration of 1000 *μ*g/mL (83.56 ± 0.06%) and EEAM at 1000 *μ*g/ml also showed a significant percentage inhibition (82.74 ± 0.68%). Aqueous extract of *Aegle marmelos* (AEAM) showed less percentage inhibition compared to Cystone. The percentage of inhibition for all extracts of *Aegle marmelos* was presented in Figure 3 and Supplementary Table S1.

All the extracts of *Drymoglossum piloselloides* showed dose-dependent inhibition on nucleation of CaOx crystals. The percentage of inhibition of ethanol extract of *Drymoglossum piloselloides* (EEDP) at 800 *μ*g/mL (71.13 ± 0.42%) and 1000 *μ*g/mL (71.13 ± 0.84%) exhibited the maximum inhibition compared to all other extracts. EEDP and hexane extract of *Drymoglossum piloselloides* (HEDP) showed higher percentage of inhibition compared to the standard drug, Cystone.

Consistent with other plant extracts, the percentage of inhibition of aqueous extract of *Drymoglossum piloselloides* (AEDP) was less than the standard drug. The percentage inhibition for all extracts of *Drymoglossum piloselloides* was presented in Figure 3 and Supplementary Table S1. Interestingly, EEDP showed the highest inhibitory effect compared to hexane and aqueous extracts although it was the hexane extract that showed the highest effect in *Kalanchoe laciniata* and *Aegle marmelos.* Based on the results obtained in this assay, the maximum percentage of inhibition was shown by HEAM at the concentration of 1000 *μ*g/mL (83.56 ± 0.06%) (Figure 3). According to the statistical analysis, there was a significant difference between the percentage inhibition of most extracts. Extracts at most all concentrations showed significant inhibitory activity compared to the positive control, Cystone, but aqueous extract of *Aegle marmelos* (AEAM) and aqueous extract of *Drymoglossum piloselloides* (AEDP) showed no significant difference in inhibition (*p* < 0.05) at the highest concentration of 1000 *μ*g/mL compared to the standard drug, Cystone.

According to the microscopic analysis, the number of COM and COD crystals formed was reduced in the presence of plant extracts and the standard drug compared to the control (Figure 3). But it was difficult to comment on the reduction of individual COM and COD crystals with the increase in concentration of extracts because some plant extracts showed a gradual inhibition with the increasing concentration and some other extracts showed a significant reduction in the crystal formation at lower concentrations.

#### 3.2.2. Growth Assay

**Figure 4:**
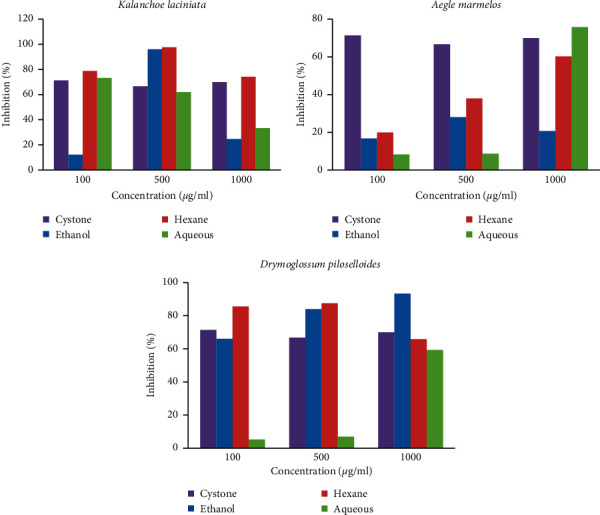
Percentage of inhibition of different extracts of plants on calcium oxalate crystal grow. Each graph shows the dose response effect of each plant extracted in ethanol, hexane, and water against the standard drug, Cystone. At any concentration of the plant extract tested, a significant difference (*p* < 0.05) in inhibition was observed between the plant extract treated tubes and the positive control, Cystone.

The inhibition of crystal growth was estimated by measuring the change in turbidity in solutions containing different concentrations of the extract relative to a control (without the extract). The extracts of *Kalanchoe laciniata* at various concentrations had an inhibitory effect on the growth of CaOx crystals, which were not dose dependent (Figure 4 and Supplementary Table S2). EEKL showed the highest percent inhibition at 500 *μ*g/mL (96.10%) and HEKL showed the maximum percent inhibition at 500 *μ*g/mL (97.69%), which were higher than the percent inhibition of Cystone at 500 *μ*g/mL (66.67%), but AEKL showed the highest inhibition at 100 *μ*g/mL (73.16%).

The percentage inhibition of growth of CaOx crystals with different extracts of *Aegle marmelos* is relatively less than other plant extracts. The highest percent inhibition was shown by AEAM at the concentration of 1000 *μ*g/mL (75.76%) that was higher than the standard drug. EEAM showed the highest inhibition at 500 *μ*g/mL (28.0%) while HEAM showed the highest inhibition at 1000 *μ*g/mL (60.19%), but it was less than the standard drug (Figure 4 and Supplementary Table S2).

Percentage of inhibition in growth in the presence of EEDP was 93.33% at the concentration of 1000 *μ*g/mL, which was the maximum inhibition shown by the extracts of *Drymoglossum piloselloides*. Therefore, the inhibitory effect on CaOx crystal growth by EEDP was higher at higher concentrations than the standard drug. On the other hand, the inhibitory effect of AEDP was very low at lower concentrations (Figure 4 and Supplementary Table S2).

The results of antiurolithiatic activity by growth assay indicated that percent inhibition was more in HEKL at 500 *μ*g/mL (97.69%), when compared with other plant extracts and it was higher than Cystone. According to the statistical analysis, at any concentration of the plant extract tested, a significant difference in inhibition was observed between the plant extract treated tubes and the positive control, Cystone.

#### 3.2.3. Aggregation Assay

The crystal aggregation was determined by the change in absorbance in the presence of extracts compared to a control without extract.

The concentrations at 200, 400, and 600 *μ*g/mL of all the extracts of *Kalanchoe laciniata* showed the percent inhibition higher than the standard drug, Cystone. At the concentrations of 800 and 1000 *μ*g/mL, percent inhibition was lower than Cystone except in HEKL. When considering percent inhibition of all extracts, HEKL had the maximum inhibition. It was 43.20 ± 1.02% (at 800 *μ*g/mL). EEKL showed maximum percent inhibition at 800 *μ*g/mL (27.49 ± 0.80%) and AEKL showed maximum percentage inhibition at 1000 *μ*g/ml (28.89 ± 1.25%).

**Figure 5:**
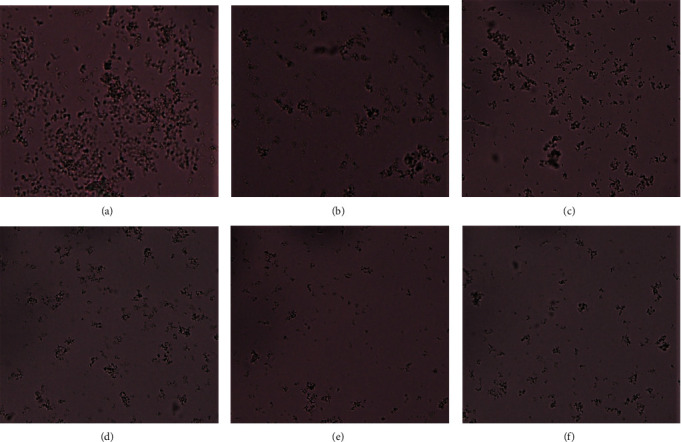
Representative micrographs of calcium oxalate crystals in the aggregation assay as observed under light microscope (x40 objective) in the absence of plant extracts (a), the CaOx crystals in aggregation assay in the presence of ethanol extract of *Aegle marmelos* (EEAM) 200 *μ*g/mL (b), 400 *μ*g/mL (c), 600 *μ*g/mL (d), 800 *μ*g/mL (e), and 1000 *μ*g/mL (f).

Inhibitory effect on aggregation of CaOx crystals was better in the presence of AEAM, which was higher at all the concentrations than Cystone. The highest inhibition was given at the concentration of 1000 *μ*g/mL (50.13 ± 1.17%). EEAM and HEAM showed inhibition of CaOx crystal aggregation higher than Cystone except at the concentrations of 800 *μ*g/mL and 1000 *μ*g/mL. Inhibitory effect of *Aegle marmelos* on aggregation of crystals exhibited a dose-dependent manner except HEAM. The highest percent inhibition of EEAM and HEAM were 36.14 ± 1.24% (at 1000 *μ*g/mL) and 31.97 ± 1.36% (at 800 *μ*g/mL), respectively. The *in vitro* inhibitory effect of ethanol extract of *Aegle marmelos* (EEAM) on various phases of CaOx aggregation as determined by the time course of turbidity measured in synthetic urine is shown in Figure 5.

**Figure 6:**
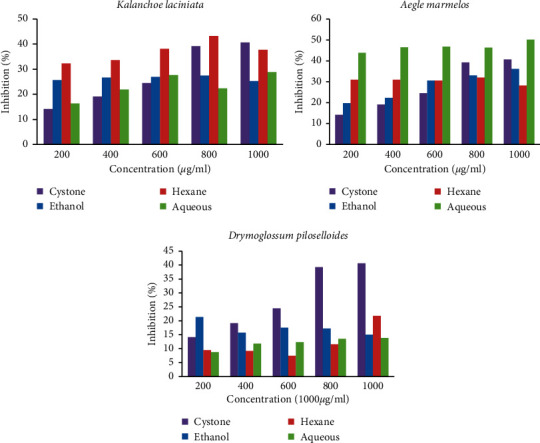
Percentage of inhibition of different extracts on CaOx crystals aggregation. Each graph shows the dose response effect of each plant extracted in ethanol, hexane, and water against the standard drug, Cystone. No significant difference in inhibition (*p* > 0.05) was observed between ethanol (at 600 *μ*g/mL), hexane (at 800 *μ*g/mL and 1000 *μ*g/mL), and aqueous (at 200 *μ*g/mL, 400 *μ*g/mL, and 600 *μ*g/mL) extracts of *Kalanchoe laciniata*, ethanol extract of *Aegle marmelos* (at 400 *μ*g/mL and 1000 *μ*g/mL) and ethanol (at 400 *μ*g/mL), hexane (at 200 *μ*g/ml), and aqueous (at 200 *μ*g/mL) extracts of *Drymoglossum piloselloides* compared to Cystone but a significant difference in percentage inhibition (*p* < 0.05) was observed in all other extracts at the concentrations shown compared to the standard drug.

The inhibitory effect on CaOx crystal aggregation was very low in all extracts of *Drymoglossum piloselloides* at any concentration when compared to the standard drug. When considering three extracts of this plant, the maximum inhibition was shown by EEDP at 200 *μ*g/mL (21.32 ± 1.50%) and HEDP at 1000 *μ*g/mL (21.77 ± 0.68%). Inhibitory effect was not dose dependent (Figure 6 and Supplementary Table S3). Depending on the results obtained in this assay, AEAM and HEKL had maximum percent inhibition against CaOx crystal aggregation. No significant difference in inhibition (*p* < 0.05) was observed between EEKL (at 600 *μ*g/mL), HEKL (at 800 *μ*g/mL and 1000 *μ*g/mL), AEKL (at 200 *μ*g/mL, 400 *μ*g/mL, and 600 *μ*g/mL), EEAM (at 400 *μ*g/mL and 1000 *μ*g/ml), EEDP (at 400 *μ*g/mL), HEDP (at 200 *μ*g/mL), and AEDP (at 200 *μ*g/mL) compared to Cystone but a significant difference in percentage inhibition (*p* < 0.05) was observed in other extracts compared to the standard drug (Figure 6 and Supplementary Table S3).

## 4. Discussion

Lithiasis, one of the causes of acute and chronic renal failure, includes urolithiasis (stone formation in ureters or bladder or both) and nephrolithiasis (stone formation in the kidney) [20]. Generally, stones are hard and crystalline minerals formed as a result of an imbalance between the inhibitors and promoters [21]. Normal urine contains many inhibitors for calculi formation (e.g., magnesium forms complexes with oxalate and reduces the supersaturation of CaOx) [22]. According to previous studies, urinary supersaturation is considered as a major contributory factor for calculi formation [23].

Renal stones are composed of mucopolysaccharide, urates, CaOx, Ca phosphate, and Ca carbonate. CaOx urolithiasis is the most prominent type of all urinary stone diseases [24]. The composition of stones varies due to many factors. CaOx crystal formation is a multistep process that includes nucleation, growth, and aggregation [25]. The present study was aimed to investigate the effect of several extracts prepared from *Kalanchoe laciniata* leaves, *Aegle marmelos* flowers, and *Drymoglossum piloselloides* leaves on the nucleation, growth, and aggregation of CaOx crystals. We used a classical model of synthetic urine supersaturated with calcium chloride and sodium oxalate to determine the effect on the three main steps in the formation of CaOx crystals.

Compared to allopathic medicine which targets one aspect of the pathophysiology of urolithiasis, plant-based drugs have shown to be effective at multiple stages in the prevention of the formation of calculi. Previous studies have reported that the phytoconstituents present in medicinal plants exert their beneficial effects by multiple mechanisms, such as changing the ionic concentration of urine, increasing the diuretic activity by increasing the volume, pH and anticalcifying activity of urine, inhibition of nucleation, growth, and aggregation during crystal formation, promoting lithotriptic activity by relieving mucin that binds calculi, and regulation of crystalloid colloid imbalance to improve renal function [26].

Nucleation is the most critical step in the process of stone formation, which begins with the combination of stone salts in the solution into loose clusters that may increase in size by adding new components [27]. Incubation of synthetic urine that contains calcium chloride and sodium oxalate results in the formation of CaOx crystals [24]. The effect of plant extracts on CaOx crystallization was evaluated by measuring turbidity of the reaction solutions compared with the control.

In the present study, nucleation of CaOx crystals was inhibited more in the presence of the three plant extracts compared to Cystone, which served as the positive control. Based on the results obtained in this study, EEAM and HEAM showed maximum inhibition and the significantly highest inhibition was shown at 1000 *μ*g/mL (EEAM, 82.74 ± 0.68% and HEAM, 83.56 ± 0.06%). A similar study conducted by Zaki et al. also reported that *Cinnamomum zeylanicum* Blume bark extract showed a similar trend in the activity at the same concentration series [24].

A study conducted previously on *in vitro* antiurolithiatic activity of methanolic and aqueous extracts of the leaves of *Peltophorum pterocarpum* revealed that both extracts showed significant antiurolithiatic activity and its activity increased proportional to the concentration. In contrast to what was reported in our study, the aqueous extract showed higher activity compared to the methanolic extract [4].

CaOx stones usually exist in two different types, the monohydrate (COM) and the dehydrate (COD). COM is considered as the thermodynamically most stable form and occur more commonly clinically with a higher affinity for renal tubular cells. Microscopic examination showed the formation of more COM crystals than COD crystals which adhere strongly to renal epithelial tissues. Therefore, COM promotes crystal retention and eventual stone formation. Furthermore, plant extracts effectively reduced the number of crystals formed compared to the control but showed lesser effect on the size of crystals [19]. Some plant extracts have the capability to transform COM crystals to COD crystals which are less likely to attach with kidney epithelial cells than COM crystals. This suggests that COD formation protects the body against stone disease because of its reduced capacity to form stable aggregates [28]. However, with the increase in the concentration of plant extracts, the presence of both COM and COD was reduced in this study and we did not observe a significant reduction of one type of crystal compared to the other. Similar to what we reported, Saha and Verma also reported a reduction in the number of crystals formed in the presence of *Bergenia ciliate rhizome extract* [29].

Crystal growth is a critical step in the urinary stone formation. Once the crystal nucleus achieves a critical size, it may grow to form a small, hard mass called a stone [30]. Therefore, crystal growth has been investigated in the presence and absence of plant extracts. From the results of this *in vitro* study, it was observed that all the plant extracts had inhibitory effect on crystal growth, but it was not dose dependent. The highest percentage inhibition on crystal growth was shown in HEKL at 500 *μ*g/ml. It was 97.69%. When considering all the plant extracts, extracts from *Aegle marmelos* showed significantly less inhibition on crystal growth compared to the standard drug, but AEAM showed a significantly higher inhibition at 1000 *μ*g/ml (75.76%).

Crystals in solution stick together to form larger particles, which is called crystal aggregation or agglomeration. It is the most important step in stone formation [27]. The aggregation was monitored in the presence and absence of plant extracts and was compared with the standard drug. The results of this *in vitro* study showed that all the plant extracts had inhibitory effect on CaOx crystal aggregation while the maximum inhibition was recorded by AEAM (50.13 ± 1.17%). At any concentration of *Aegle marmelos,* it showed a better inhibition on crystal aggregation which was significantly higher than the standard drug. These results were compatible with the results reported by Saha and Verma [29].

According to data reported previously, the inhibitory effect of the extract of *Kalanchoe pinnata*, a different species of the plant that we studied, has also been investigated *in vitro* on nucleation and aggregation of crystals. Contrary to what was reported in our study, this study has revealed that the extract had greater capability to dissolve CaOx while Cystone, the standard, had shown better demineralization for calcium phosphate than the plant extract. This extract had exhibited inhibitory activity in both nucleation and aggregation assays to a significant level [31]. Another study conducted by Sohgaura et al. reported that *K. pinnata* had high content of total flavonoid and polyphenols, which was supported by its potential in vitro in CaOx crystal dissolution and crystal growth inhibition [32]. Another study was conducted on the polyherbal tablets containing *K. pinnata* extracts as one of the constituents that were found effective against the accumulation of CaOx crystals and prevention of stone formation within the kidneys [33].

Qualitative phytochemical screening of *Kalanchoe laciniata* revealed the presence of reducing sugars, proteins, flavonoids, tannins, and polyphenol compounds while *Aegle marmelos* showed the presence of reducing sugars, proteins, anthracene glycosides, and saponins. Preliminary phytochemical screening of *Drymoglossum piloselloides* showed the presence of reducing sugars, proteins, flavonoids, tannins, and polyphenol compounds. Saponins are known to have anticrystallization properties by disintegrating mucoproteins and as promoters of crystallization [29] and their inhibitory effect in urolithiasis has already been reported previously using plants such *Solanum xanthocarpum* [34], *Trachyspermum ammi* [35], and *Achyranthes aspera* [36]. Flavonoids possess CaOx crystal dissolution property and tannins and polyphenols inhibit CaOx crystal formation and dissolve the preformed CaOx crystals [19].

Using quantitative analysis, it was reported previously by several authors that polyphenols and flavonoids are present in significant quantities in all three plants under study [37–40]. In comparison to what was reported in this study, a comprehensive analysis of phytochemicals of *Aegle marmelos* was reported by Lambole et al. [41]. Different parts of the tree are rich in different phytochemicals including alkaloids, terpenoids, and coumarins, such as scoparone, scopoletin, umbelliferone, and marmesin [42, 43]. Previous studies also provided evidence that *Aegle marmelos* also contain bioactive compounds, such as xanthotoxol, imperatorin, aegeline, and marmeline and the unique fatty acid, 12-hydroxyoctadec-cis-9-enoic acid, or ricinoleic acid. These compounds provide antidiabetic, anticancer, antifertility, antimicrobial, immunogenic, analgesic, and insecticidal activities but none of the studies reported antiurolithiatic potential of the plant [44]. A study conducted on leaves of *Drymoglossum piloselloides* collected from Kurunegala in Sri Lanka detected unsaturated sterols, polyphenolic compounds, and flavonoids as the phytochemicals of interest [45], very similar to what was reported in this study. According to Dalimartha, sterols/triterpenes, phenols, flavonoids and tannin were reported in *Drymoglossum piloselloides* as major constituents in a study conducted in India [46]. Among the constituents that have been identified so far in *Kalanchoe laciniata*, flavonoids represent the class of secondary metabolites most commonly found. In addition, some patuletin aglycone derivatives, carotenoids and polysaccharides, have also been identified. A majority of the studies indicated the presence of glycosylated flavonoids, derived from the acetylation of the rhamnose ring of patuletin in different positions [47].

Several medicinal plant extracts have been reported for *in vitro* antiurolithiatic activity worldwide. But, to date, scientific pieces of evidence supporting *in vitro* antiurolithiatic effect of *Kalanchoe laciniata*, *Aegle marmelos*, and *Drymoglossum piloselloides* have not been reported. However, scientific pieces of evidence for another species of this plant, *Kalanchoe pinnata*, have been reported previously. That study was conducted using only an aqueous extract of *Kalanchoe pinnata* leaves, which showed a better inhibition on crystal nucleation and aggregation. But we have proven that three different extracts of *Kalanchoe laciniata* have inhibitory effects on crystal nucleation, aggregation, and crystal growth. Phytoconstituents present in these two species are also different. Activity-guided fractionation and *in vivo* studies need to be carried out to identify the compounds responsible for the activity and for a better understanding of molecular mechanisms of lithiasis in the future.

A limitation of the study was the inability to quantify the main contributors of the antiurolithiatic mechanism of *in vitro* systems, citric acid concentration, and magnesium levels, which will be investigated in the future. The mechanism of action of the extracts in animal models of lithiasis needs to be investigated in the future for a detailed understanding of the mechanism of action of the plant extracts used in the study.

## 5. Conclusion

The present study demonstrates a significant antiurolithiatic potential of the three selected medicinal plants having traditional claims in ayurvedic medicine against CaOx urolithiasis *in vitro*. Phytoconstituents such as saponins, tannins, and flavonoids, responsible for antiurolithiatic activity, were present in the plants used in this study. This study has given primary evidence for *Kalanchoe laciniata*, *Aegle marmelos*, and *Drymoglossum piloselloides* as the plants which possess significant antiurolithiatic property. *Aegle marmelos* showed better inhibition at 1000 *μ*g/mL on CaOx crystal nucleation compared to other plants. When considering extracts of *Aegle marmelos*, ethanol and hexane extracts showed better inhibition on crystal nucleation. Water extract of *Aegle marmelos* showed better inhibition at 1000 *μ*g/mL on crystal aggregation compared to all the extracts. Ethanol and hexane extracts of *Kalanchoe laciniata* showed highest inhibition on crystal growth. But aqueous extract of *Aegle marmelos* also showed high percentage of inhibition on crystal growth at 1000 *μ*g/mL. So, this study has proven that *Aegle marmelos* has better antiurolithiatic potential compared to other two plants.

## Figures and Tables

**Table 1:** Phytoconstituents identified by the qualitative assays in the three plants under study.

Phytoconstituents	Plant species		
	*Kalanchoe laciniata*	*Aegle marmelos*	*Drymoglossum piloselloides*
Reducing sugars	+	+	+
Proteins	+	+	+
Flavonoids	+	−	+
Tannins	+	−	+
Polyphenol compounds	+	−	+
Anthracene glycosides	−	+	−
Saponins	−	+	−
Cyanogenic glycosides	−	−	−
Alkaloids	−	−	−

(+) indicates presence whereas (−) indicates absence of phytoconstituents.

## Data Availability

The datasets used and/or analyzed during the current study are available from the corresponding author upon reasonable request.

## Supplementary Materials

Supplementary Materials Table S1: antiurolithiatic activity of plant extracts compared to Cystone as determined by the nucleation assay. Table S2: antiurolithiatic activity of plant extracts compared to Cystone as determined by the growth assay. Table S3: antiurolithiatic activity of plant extracts compared to Cystone as determined by the aggregation assay.
