# Supplementary material for: Antiurolithiatic Potential of Three Sri Lankan Medicinal Plants by the Inhibition of Nucleation, Growth, and Aggregation of Calcium Oxalate Crystals In Vitro
Source: ScientificWorldJournal. 2022 Apr 12;2022:8657249. doi: 10.1155/2022/8657249 (PMC9019441; doi:10.1155/2022/8657249)
Supplement: Supplementary Materials — Table S1: antiurolithiatic activity of plant extracts compared to Cystone as determined by the nucleation assay. Table S2: antiurolithiatic activity of plant extracts compared to Cystone as determined by the growth assay. Table S3: antiurolithiatic activity of plant extracts compared to Cystone as determined by the aggregation assay. [file 8657249.f1.docx]

**Supplementary data**

**Table S1:** Antiurolithiatic activity of plant extracts compared to cystone as determined by the

nucleation assay

| **Concentrations (µg/ml)** | **Standard/ Plant extracts / % inhibition ± SEM** | | |
| --- | --- | --- | --- |
| Cystone |  | | |
|  |  | | |
| 200 | 20.92 ±0.26 | | |
| 400 | 21.49 ±0.21 | | |
| 600 | 21.54 ±0.15 | | |
| 800 | 21.44 ±0.67 | | |
| 1000 | 18.39 ±0.93 | | |
|  |  |  |  |
| ***Kalanchoe laciniata*** | **EEKL** | **HEKL** | **AEKL** |
|  |  |  |  |
| 200 | 25.69 ±0.15 * | 37.53 ±0.25 * | 13.79 ±0.31 * |
| 400 | 33.41 ±0.51 * | 43.45 ±0.88 * | 15.50 ±0.55 * |
| 600 | 39.87 ±0.07 * | 47.23 ±0.50 * | 13.42 ±0.55 * |
| 800 | 41.56 ±0.44 * | 57.93 ±0.50 * | 14.09 ±0.00 * |
| 1000 | 47.87 ±0.29 * | 59.01 ±0.31 * | 12.93 ±0.31 * |
|  |  |  |  |
| ***Aegle marmelos*** | **EEAM** | **HEAM** | **AEAM** |
|  |  |  |  |
| 200 | 39.27 ±0.68 * | 44.21 ±0.25 * | 10.17 ±0.25 * |
| 400 | 45.65 ±0.00 * | 53.09 ±0.94 * | 12.07 ±0.06 * |
| 600 | 56.39 ±0.95 * | 63.66 ±1.07 * | 14.28 ±0.06 * |
| 800 | 69.97 ±0.95 * | 78.34 ±0.38 * | 15.20 ±0.24 * |
| 1000 | 82.74 ±0.68 * | 83.56 ±0.06 * | 18.26 ±0.61 |
|  |  |  |  |
| ***Drymoglossum piloselloides*** | **EEDP** | **HEDP** | **AEDP** |
|  |  |  |  |
| 200 | 46.23± 0.21 * | 37.34± 0.31 * | 16.24± 0.18 * |
| 400 | 61.09± 1.26 * | 39.61± 0.57 * | 16.48± 0.18 * |
| 600 | 62.34± 0.84 * | 41.88± 0.31 * | 17.83± 0.43 * |
| 800 | 71.13± 0.42 * | 40.43± 0.76 * | 17.03± 0.12 * |
| 1000 | 71.13± 0.84 * | 43.77± 0.06 * | 18.20± 0.43 |

Values are expressed as mean ± SEM, * Significant compared to Cystone, p < 0.05. Ethanol extract of *Kalanchoe laciniata* (EEKL),hexane extract of *Kalanchoe laciniata* (HEKL), aqueous extract of *Kalanchoe laciniata* (AEKL), Ethanol extract of *Aegle marmelos* (EEAM), hexane extract of *Aegle marmelos* (HEAM), Aqueous extract of *Aegle marmelos* (AEAM), Ethanol extract of *Drymoglossum piloselloides* (EEDP), hexane extract of *Drymoglossum piloselloides* (HEDP), Aqueous extract of *Drymoglossum piloselloides* (AEDP)

**Table S2:** Antiurolithiatic activity of plant extracts compared to cystone as determined by the growth assay

| **Concentrations (µg/ml)** | **Standard / Plant extracts / % inhibition ± SEM** | | |
| --- | --- | --- | --- |
| Cystone |  | | |
|  |  | | |
| 100 | 71.30 | | |
| 500 | 66.67 | | |
| 1000 | 69.91 | | |
|  |  |  |  |
| ***Kalanchoe laciniata*** | **EEKL** | **HEKL** | **AEKL** |
|  |  |  |  |
| 100 | 12.12 ± 0.24* | 78.70 ± 0.45* | 73.16 ± 0.66* |
| 500 | 96.10 ± 0.32 * | 97.69 ± 1.02* | 61.90 ± 0.84* |
| 1000 | 24.68 ± 0.35* | 74.07 ± 0.65* | 33.33 ± 0.54* |
|  |  |  |  |
| ***Aegle marmelos*** | **EEAM** | **HEAM** | **AEAM** |
|  |  |  |  |
| 100 | 16.67 ± 0.65* | 19.91 ± 0.23* | 8.23 ± 0.21* |
| 500 | 28.00 ± 0.95* | 37.96 ± 0.32* | 8.66 ± 0.22* |
| 1000 | 20.67 ± 0.33* | 60.19 ± 0.42* | 75.76 ± 0.84* |
|  |  |  |  |
| ***Drymoglossum piloselloides*** | **EEDP** | **HEDP** | **AEDP** |
|  |  |  |  |
| 100 | 66.00 ± 0.88* | 85.65 ± 0.98* | 5.19 ± 0.09* |
| 500 | 84.00 ± 0.75* | 87.50 ± 0.87* | 6.93 ± 0.08* |
| 1000 | 93.33 ±1.21* | 65.74 ± 0.77* | 59.31 ± 0.54* |

Values are expressed as mean ± SEM, * Significant compared to Cystone, p < 0.05. Ethanol extract of *Kalanchoe laciniata* (EEKL),hexane extract of *Kalanchoe laciniata* (HEKL), aqueous extract of *Kalanchoe laciniata* (AEKL), Ethanol extract of *Aegle marmelos* (EEAM), hexane extract of *Aegle marmelos* (HEAM), Aqueous extract of *Aegle marmelos* (AEAM), Ethanol extract of *Drymoglossum piloselloides* (EEDP), hexane extract of *Drymoglossum piloselloides* (HEDP), Aqueous extract of *Drymoglossum piloselloides* (AEDP)

**Table S3:** Antiurolithiatic activity of plant extracts compared to cystone as determined by the aggregation assay

| **Concentrations (µg/ml)** | **Standard / Plant extracts / % inhibition ± SEM** | | |
| --- | --- | --- | --- |
| Cystone |  | | |
|  |  | | |
| 200 | 14.14 ±1.79 | | |
| 400 | 19.12 ±0.80 | | |
| 600 | 24.50 ±1.00 | | |
| 800 | 39.24 ±1.00 | | |
| 1000 | 40.64 ±0.40 | | |
|  |  |  |  |
| ***Kalanchoe laciniata*** | **EEKL** | **HEKL** | **AEKL** |
|  |  |  |  |
| 200 | 25.70 ± 0.60 * | 32.31 ± 1.02 * | 16.33 ± 0.75 |
| 400 | 26.69 ± 0.80 * | 33.67 ± 0.34 * | 21.86 ± 0.25 |
| 600 | 26.89 ± 1.40 | 38.10 ± 0.68 * | 27.64 ± 1.00 |
| 800 | 27.49 ± 0.80 * | 43.20 ± 1.02 | 22.36 ± 1.25 * |
| 1000 | 25.30 ± 1.00 * | 37.76 ± 1.02 | 28.89 ± 1.25 * |
|  |  |  |  |
| ***Aegle marmelos*** | **EEAM** | **HEAM** | **AEAM** |
|  |  |  |  |
| 200 | 19.78 ± 0.16 * | 30.95 ± 0.34 * | 43.88 ± 0.91 * |
| 400 | 22.27 ± 0.78 | 30.95 ± 1.02 * | 46.48 ± 0.65 * |
| 600 | 30.53 ± 0.62 * | 30.61 ± 0.68 * | 46.74 ± 0.91 * |
| 800 | 33.02 ± 0.62 * | 31.97 ± 1.36 * | 46.35 ± 0.78 * |
| 1000 | 36.14 ± 1.25 | 28.23 ± 1.02 | 50.13 ± 1.17 * |
|  |  |  |  |
| ***Drymoglossum piloselloides*** | **EEDP** | **HEDP** | **AEDP** |
|  |  |  |  |
| 200 | 21.32 ±1.50 * | 9.52 ± 0.68 | 8.79 ± 0.75 |
| 400 | 15.77 ±1.35 | 9.18 ± 1.02 * | 11.81 ± 1.26 * |
| 600 | 17.57 ± 1.35 * | 7.48 ± 1.36 * | 12.31 ± 0.75 * |
| 800 | 17.27 ± 1.35 * | 11.56 ± 1.36 * | 13.57 ± 1.00 * |
| 1000 | 15.02 ± 0.60 * | 21.77 ± 0.68 * | 13.82 ± 0.75 * |

Values are expressed as mean ± SEM, * Significant compared to Cystone, p < 0.05. Ethanol extract of *Kalanchoe laciniata* (EEKL),hexane extract of *Kalanchoe laciniata* (HEKL), aqueous extract of *Kalanchoe laciniata* (AEKL), Ethanol extract of *Aegle marmelos* (EEAM), hexane extract of *Aegle marmelos* (HEAM), Aqueous extract of *Aegle marmelos* (AEAM), Ethanol extract of *Drymoglossum piloselloides* (EEDP), hexane extract of *Drymoglossum piloselloides* (HEDP), Aqueous extract of *Drymoglossum piloselloides* (AEDP)
